# Nucleostemin- and Oct 3/4-positive stem/progenitor cells exhibit disparate anatomical and temporal expression during rat Achilles tendon healing

**DOI:** 10.1186/s12891-015-0658-3

**Published:** 2015-08-20

**Authors:** Eva Runesson, Paul Ackermann, Jón Karlsson, Bengt I Eriksson

**Affiliations:** Department of Orthopaedics, Lundberg Laboratory for Orthopaedic Research, Institute of Clinical Sciences, University of Gothenburg, Gröna Stråket 12, Sahlgrenska University Hospital, SE-413 45 Gothenburg, Sweden; Integrative Orthopaedic Laboratory, Department of Molecular Medicine and Surgery, Karolinska Institutet, Stockholm, Sweden

## Abstract

**Background:**

The recent discovery of residing tendon stem/progenitor cells has triggered a growing interest in stem cells as a useful tool in tendon repair. Our knowledge of their involvement in naturally healing tendons is, however, sparse. The aim of this study was to identify and determine stem/progenitor cells in relation to different healing phases and regions in a rat model of Achilles tendon rupture.

**Methods:**

Surgery was performed to create a mid-tendon rupture on the right Achilles tendon of 24 rats, whereas the left tendon was used as a control. Tendons were harvested at one, two, eight and 17 weeks post-rupture and stained with antibodies specific to stem/progenitor cells (Octamer-binding transcription factor 3/4 (Oct 3/4) and nucleostemin), migrating cells (Dynamin 2 (Dyn 2)) and leukocytes (CD45). A histological examination was performed on sections stained with Alcian blue.

**Results:**

At one and two weeks post-rupture, a large number of stem/progenitor cells were discovered throughout the tendon. Most of these cells were nucleostemin positive, whereas only a few Oct 3/4-positive cells were found, mainly situated inside the injury region (I region). At eight and 17 weeks, the increment in stem/progenitor cells had diminished to equal that in the control tendons. At all time points, Oct 3/4-positive cells were also found in the connective tissue surrounding the tendon and at the muscle-tendon junction in both ruptured and control tendons and were often seen at the same location as the migration marker, Dyn 2.

**Conclusions:**

The whole length of the Achilles tendon is infiltrated by stem/progenitor cells at early time points after a mid-tendon rupture. However, different stem/progenitor cell populations exhibit varying anatomical and temporal expressions during Achilles tendon healing, suggesting distinct reparative implications. Oct 3/4 may thus act as a more local, migrating stem/progenitor cell involved in injury-site-specific regenerative effects, as compared to the more general proliferative role of nucleostemin-positive stem/progenitor cells.

## Background

Clinically, there is as yet no treatment/method to speed up or improve tendon repair in order finally to re-create a fully functional tendon. The tendon healing process is commonly described in three phases. The inflammatory phase (approximately 1–2 weeks), a short period of massive influx of erythrocytes and inflammatory cells, is followed by a reparative phase (approximately 2–6 weeks), in which the proliferation and migration of both intrinsic and extrinsic cells appear to occur [1, 2]. The reparative phase is followed by a remodelling phase (approximately 4- weeks) in order to stabilise, align and mature the tendon structure [3]. Unfortunately, despite its ability to heal, a ruptured tendon never attains the same biochemical properties or mechanical strength as an intact tendon [4–6]. The discovery of tendon-specific stem/progenitor cells [7] has stimulated research on the opportunity to utilise these stem/progenitor cells to promote tendon healing.

However, the way the residing tendon stem/progenitor cells influence tendon healing processes is still partially unknown and it is therefore important to obtain a better understanding of the regulatory mechanisms in tendon repair. Tendon stem/progenitor cells have been found to exist in several different regions in and around the tendon tissue, possibly with various influences on the healing tendon tissue [8–11]. Recent studies have demonstrated a residing stem cell pool in the distal region of rat Achilles and patellar tendons, in a higher proportion than in the mid-tendon region [10, 11]. Moreover, the peritendon region appears to harbour stem/progenitor cells, possibly involved in both tendon homeostasis and tendon repair processes [8, 9]. It has further been found that stem/progenitor cells originating from regions within and around tendon tissue have both similar and dissimilar properties, indicating a dual mechanism for tendon healing (intrinsic and/or extrinsic) [8, 9]. The stem/progenitor cells from the peritendon, as well as the tendon proper, could be driven towards osteogenic differentiation, possibly leading to undesirable ectopic tissue during tendon healing [8, 9]. Furthermore, it has been found that the progenitor cells originating from the tendon proper have greater potential for forming tendon-like tissue compared with the progenitor cells originating from the tissue surrounding the tendon [12]. Most studies of progenitor cells involved in tendon healing have used *in-vitro* methods [12–14] or have mainly been performed on regions in or around the injury [11]. The identification of regional and temporal differences in stem/progenitor cells in the natural *in-vivo* tendon healing processes may provide crucial information when it comes to selecting the best strategies for the internal activation of tendon regeneration and healing.

In the present study, we used a rat Achilles tendon rupture model to study histological tendon morphology, stem/progenitor cells and migration during early and late tendon repair in different regions of the whole length of the Achilles tendon. It was hypothesised that, after a mid-Achilles tendon injury, different stem/progenitor cells would exhibit differential regional distribution and time-dependent expression during healing.

## Methods

### Animals

Twenty-four female Sprague–Dawley rats (Charles River, Germany) with an initial weight of 195–221 g (median = 205 g) were included in the study. The animals were acclimatised and housed three per cage in a 12-hour dark/light cycle for one week prior to any surgical procedure. The animals were housed with free access to food and water and all the experiments were approved by the Gothenburg Ethical Committee on Animal Research (Dnr: 257–2009).

### Surgery model

Prior to the surgical procedure, the rats were anaesthetised with an isoflurane inhalation (Baxter Medical AB, Sweden) in a cage, transferred to an aseptic clean bench and connected to a breathing mask with a constant flow of isoflurane. Analgesic treatment was performed once pre- and post-operatively with a subcutaneous injection of Temgesic (0.3 mg/ml, Schering-Plough, Sweden). A 1 cm longitudinal incision was made lateral to the right Achilles tendon. The Achilles tendon complex was exposed and the plantaris tendon was gently moved aside. A plain forceps was slid under the Achilles tendon and a complete rupture was performed by tearing the collagen fibres apart using a Ewald teeth forceps (TG instrument, Helsingborg, Sweden). The rupture was made approximately 0.5 cm from the calcaneal insertion, carefully leaving the plantaris tendon undamaged. The skin was sutured and the rats were allowed unrestricted movement in their cages. In all animals, the Achilles tendon from the contralateral left limb was left intact and served as a control. During the first week post-surgery, rats were monitored daily for signs of pain, stress or infection. There were no animal drop-outs during the experiment and the rats were euthanised with a lethal intraperitoneal dose of sodium pentobarbital (Apoteket Production & Laboratorier AB (APL), Sweden) in groups of six at time points one, two, eight and 17 weeks post-surgery.

### Histology and immunohistochemistry

At harvest, the Achilles tendon complex, including both the calcaneal bone and parts of the gastrocnemius muscle, from both the right (ruptured) and left (control) tendons, was put in Histofix solution (Histolab Products AB, Göteborg, Sweden) for 24 h at 4 °C, followed by decalcification in 0.5 M EDTA/0.5 % paraformaldehyde solution for a period of two weeks, washed, dehydrated and embedded in paraffin. Serial longitudinal sections in the coronal plane (5–6 μm) were cut on a microtome and mounted on plus-slide glasses (Histolab Products AB, Göteborg, Sweden). After deparaffinisation, sections were stained with Alcian blue/Van Gieson/HTX to obtain a histological overview of the tendon samples.

Immunofluorescence was performed on tendon sections using the stem/progenitor cell markers, nucleostemin (1/100, polyclonal GT 15050, Neuromics, Edina, USA) and Oct 3/4 (1/50, monoclonal sc-5279, Santa Cruz Biotech, Santa Cruz, CA), the migration/endocytosis marker, Dynamin 2 (1/100, polyclonal ab 3457, Abcam, Cambridge, UK), and the common leukocyte antigen, CD45 (1/100, monoclonal MCA43GA, AbD Serotec, Oxford, UK).

Sample sections were deparaffinised, followed by a heat-induced antigen retrieval step in HIER-EDTA pH 9.0 (used for nucleostemin and Oct 3/4, Nordic Biosite, Täby, Sweden) or HIER-Citrate pH 6.0 (used for Dynamin 2 and CD 45, Nordic Biosite, Täby, Sweden). Sections were permeabilised in 0.1 % Triton X-100, washed, blocked with Image-iT® FX Signal Enhancer (Life Technologies, Carlsbad, CA) for 20 min and incubated with primary antibodies over night at 4 °C. On the following day, the sections were washed and incubated with Alexa-Fluor conjugated secondary antibodies (goat anti-rabbit 488 used for Dynamin 2, donkey anti-goat 594 used for nucleostemin, goat anti-mouse 546 used for Oct 3/4 and goat anti-mouse 594 for CD45) for three hours at RT. The samples were covered with ProLong® Gold Antifade Reagent with DAPI (Life Technologies, Carlsbad, CA). Positive control tissue came from human seminoma (for stem cell markers nucleostemin and Oct 3/4), rat spleen (for CD 45) and rat thymus (for Dynamin 2). Negative controls were derived from the original samples with the exclusion of the primary antibody. Sections were examined using a Nikon fluorescence microscope (Eclipse E600, Nikon, Tokyo, Japan).

### Histological analysis

**Fig. 1:**
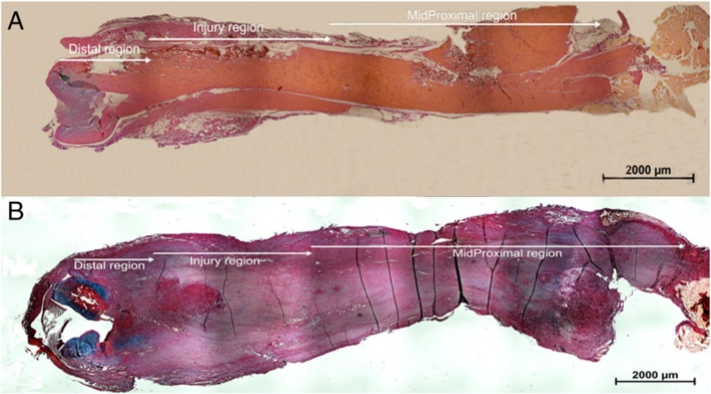
Definition of tendon regions. Sections of whole rat Achilles tendon in the coronal plane from control (**a**) and ruptured (**b**) rat Achilles tendon were used to define the distal (D), injury (I) and midproximal (MP) regions. Staining = Alcian blue/Van Gieson

Three regional areas (distal region (D), injury region (I) and midproximal region (MP)) were defined, from both the right ruptured tendon and the left contralateral tendon, and stained with Alcian blue/Van Gieson. The distal region was set at a length of ~2 mm (excluding the calcified fibrocartilage zone), the injury region at a length of ~4 mm and the midproximal region as all the parts of the tendon above the injury region to the muscle-tendon insertion (Fig. 1). Visual histological evaluations of Alcian blue/Van Gieson and fluorescence staining were performed in the different regions at all time points. The cell density (cells/mm^2^), nucleostemin density (nucleostemin-positive cells/mm^2^) and percentage of cells positive for nucleostemin were further calculated in all parts of the tendon at all time points. Furthermore, calculations of nucleostemin-positive cells in injured tendons at 17 weeks post-rupture were performed in five different regions, distal region (D), injury region (I) (with no visible Alcian blue staining), midproximal region (MP) (with no visible Alcian blue staining), surrounding connective tissue region (CT) and regions inside the tendon proper containing strong Alcian blue staining (AB) (Alcian blue islands).

### Image calculation

To calculate the cell density, nucleostemin-stained cell density and percentage of nucleostemin-positive cells, images were captured along the whole tendon, excluding only the parts with visible damage and low-integrity structures due to sample preparation. One slide from each sample was used for image analysis and all exposures were performed at the same settings. All images for calculation purposes were captured under a 20X objective, giving an image view of 0.6 mm x 0.46 mm (for all views). From each time point, six ruptured and six control (contralateral tendon) tendons were calculated. In each view, an automatic calculation was made with the NIS-element software (NIKON, Tokyo, Japan). First, threshold levels were defined for blue (DAPI) and red fluorescence (nucleostemin) and they were used in all calculations. Automatic calculations were then made on DAPI/nucleostemin merged pictures using “counting object” as the calculation setting. The total number of DAPI-stained cells/mm^2^ and the number of nucleostemin-positive cells/DAPI-stained cells were recorded and presented as the total number of cells/mm^2^, nucleostemin-positive cells/mm^2^ and the percentage of nucleostemin-positive cells.

To calculate the percentage of nucleostemin-positive cells in injured tendons at 17 weeks post-rupture, three representative areas of approximately 0.6 mm x 0.46 mm were selected from the five different regions. Counting was performed manually on DAPI/nucleostemin merged images and calculated as the percentage of nucleostemin-positive cells.

### Statistics

Data are presented with the median and interquartile range (IQR). The Kruskal-Wallis test (more than two independent groups) was used when studying possible time-dependent differences in total cells/mm^2^, nucleostemin-stained cells/mm^2^ and the percentage of nucleostemin-positive cells, followed by the Mann–Whitney *U*-test (post-hoc, two independent groups). The Mann–Whitney *U*-test was used to test for differences between control and ruptured tendon at different time points. The same statistical method was performed when the percentages of nucleostemin-positive cells in different regions of injured rat Achilles tendon 17 weeks post-rupture were compared. All analyses were performed using SPSS software (IBM, SPSS version 19.0) and statistical significance was set at p < 0.05.

## Results

### Tissue morphology

**Fig. 2:**
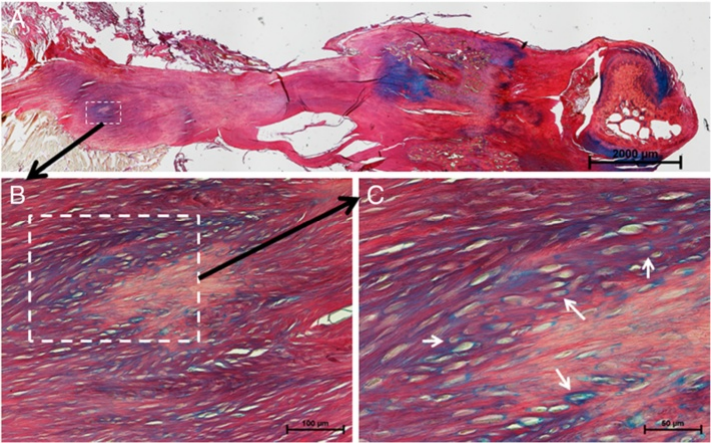
Histology of healing tendon at week 17 post-rupture. A residing island of Alcian blue-positive proteoglycan matrix was seen in the MP and I regions of late healing tendon (**a**). An enlargement of the dashed white box in (**a**) shows an Alcian blue-positive area in the MP region (**b**), where further magnification shows rounded chondrocyte-like cells (white arrows) in this area (**c**)

Areas of proteoglycan-producing cells, as detected by Alcian blue, in all control tendons were only found in the distal (D) region and there were no visible changes over time. The ruptured tendons displayed a different location for the Alcian blue staining as compared to the controls. At weeks one and two post-rupture, weak Alcian blue staining was observed, equally distributed inside the whole length of the Achilles tendon. At week 17 post-rupture, distinct islands of Alcian blue staining were seen in the injury (I) and mid/proximal (MP) regions of the ruptured tendon (Fig. 2). Cells in and around these Alcian blue islands had rounded shapes more similar to chondrocytes (Fig. 2). As a result, during the observation period 17 weeks post-rupture, the morphology did not return to normal.

### Immunohistological analyses of Oct 3/4-, Dynamin 2- and CD 45-positive cells

**Fig. 3:**
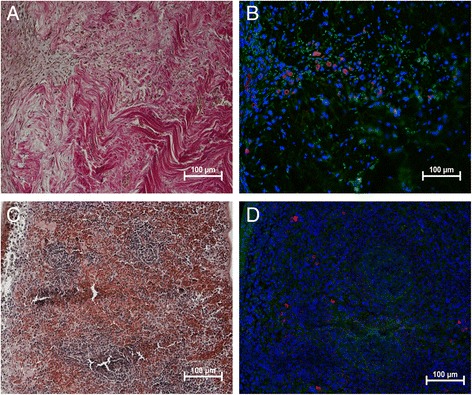
Leukocytes in early tendon healing. Photomicrographs show the histological staining (**a**) and adjacent consecutive sections of CD 45 (red) (common leukocyte antigen) staining in rat tendon (**b**) from one week post-rupture. Positive antibody control tissue was rat spleen (**c**,**d**). Cell nuclei stained with DAPI (blue). Images are merged with green auto-fluorescence to make the red blood cells (green) visible

Few CD 45-positive cells in both control and ruptured tendons could be observed in the close vicinity of blood vessels located in the connective tissue surrounding the tendon. No CD 45-positive cells were seen inside any of the regions of the control tendons. In the ruptured tendons, however, at one and two weeks post-rupture, some CD 45-positive cells were found in the I region (Fig. 3).

**Fig. 4:**
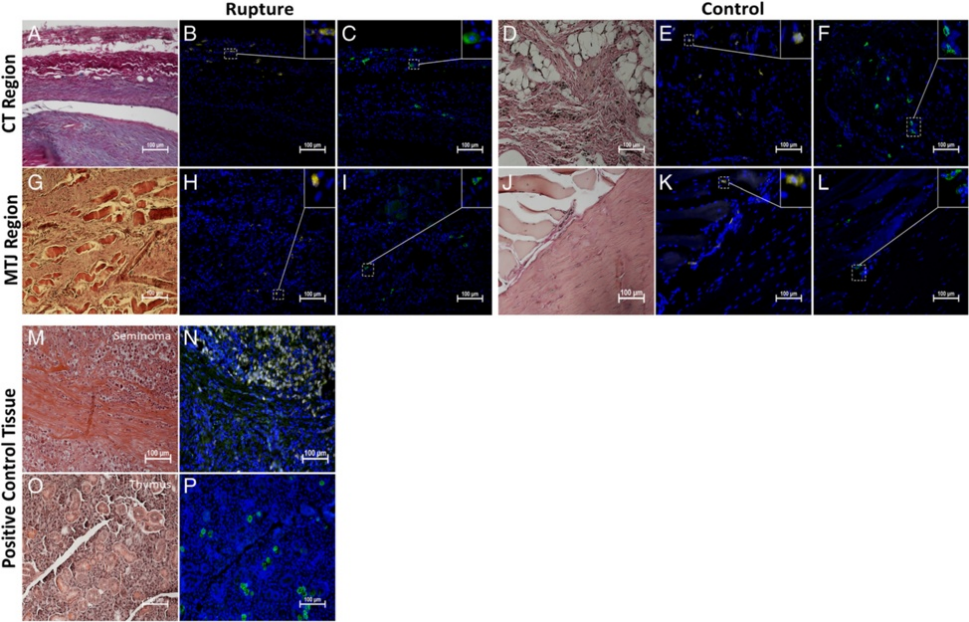
Dynamin 2 and Oct 3/4 in CT and MTJ region in rat Achilles tendon. Photomicrographs show the histological staining and adjacent consecutive sections of Oct 3/4 (yellow) and Dyn 2 (green) staining in ruptured and control tendons from the CT region (**a**-**f**) and MTJ region (**g**-**l**). Positive cells for Oct 3/4 and Dyn 2 was seen in both ruptured and control tendons in both regions. All tendon samples are from one or two weeks post-rupture. Positive control tissue for Oct 3/4 staining was human seminoma tissue (**m**,**n**) and rat thymus for Dyn 2 (**o**,**p**). Cell nuclei stained with DAPI (blue)

**Fig. 5:**
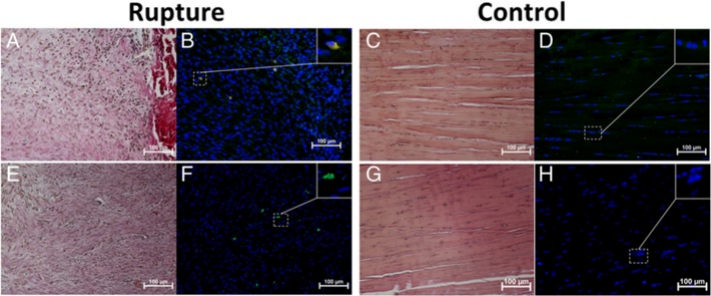
Dynamin 2 and Oct 3/4 in I region in early tendon healing. Sections showing few positive Oct 3/4 (yellow) (**b**) and Dyn 2 (green) (**f**) cells inside the I region of Achilles rat tendon at one week post-rupture. No Oct 3/4- (**d**) or Dyn 2- (**h**) -positive cells were found inside the I region of control tendons (contralateral intact tendon). Histological staining of sections of ruptured (**a**,**e**) and control (**c**,**g**) sections was performed on adjacent consecutive sections. Cell nuclei stained with DAPI (blue)

Dynamin 2-positive cells were found in control and ruptured tendons both in the connective tissue surrounding the tendon (Fig. 4) and at the muscle-tendon junction (Fig. 4). At the early time points post-rupture (1 and 2 weeks), some Dynamin 2-positive cells were observed deeper inside the I region, whereas no Dynamin 2-positive cells were seen inside the tendon proper of the control tendons (Fig. 5).

Like Dynamin 2, Oct 3/4-positive cells were found in control and ruptured tendons in the loose connective tissue surrounding the tendon (Fig. 4) and at the tendon-muscle junction at all time points (Fig. 4). Inside the ruptured tendons, Oct 3/4-positive cells could be seen in the I region at weeks one and two post-rupture (Fig. 5). At week 17 post-rupture, a few Oct 3/4-positive cells were observed inside the tendon and they were mainly located in the vicinity of blood vessel streaks. In control tendons, no Oct 3/4-positive cells were found in the tendon proper at any time point (Fig. 5).

### Cell density in the ruptured vs. contralateral control tendon

**Fig. 6:**
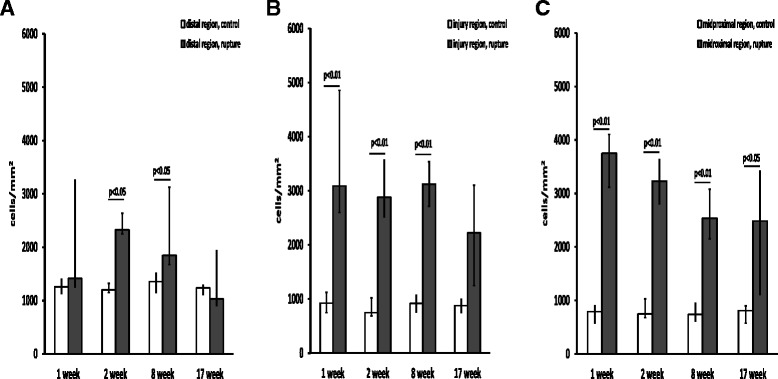
Cell density in ruptured vs contralateral control tendon. Total cell numbers/mm^2^ in (**a**) the distal region, (**b**) the injury region and (**c**) the midproximal region at one, two, eight and 17 weeks post-rupture. A total of six animals were evaluated at each time point. The *p* values in the figure represent comparisons between the control (left leg *n* = 6) and ruptured (right leg *n* = 6) tendon samples at each time point. Values are expressed as the median with IQR as error bars

In the I and MP regions of the ruptured tendons, the cell density (~2,500-4,000 cells/mm^2^) was approximately three times higher compared with the control tendons (~850-950 cells/mm^2^) at all time points (I region p < 0.01 weeks 1–8; MP region p < 0.01 weeks 1–8 and p < 0.05 week 17) (Fig. 6). In the D region, however, the cell density in the ruptured tendons (1,500-2,000 cells/mm^2^) was only significantly higher at two and eight weeks post-injury as compared with the controls (~1,200 cells/mm^2^) (Fig. 6).

### Nucleostemin cell density in the ruptured vs. contralateral control tendon

**Fig. 7:**
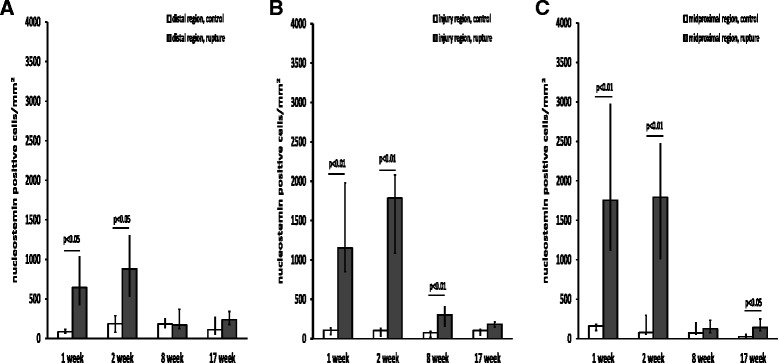
Nucleostemin cell density in ruptured vs contralateral control tendon. Nucleostemin-positive cells/mm^2^ in the distal (**a**), injury (**b**) and midproximal (**c**) regions at one, two, eight and 17 weeks post-rupture. A total of six animals were evaluated at each time point. The *p* values in the figure represent comparisons between the control (left leg *n* = 6) and ruptured (right leg *n* = 6) tendon at each time point. Values are expressed as the median with IQR as error bars

The density of nucleostemin-positive cells at weeks one and two post-injury was significantly higher in all regions of ruptured tendons when compared with control tendons. The highest nucleostemin cell density at weeks one to two was observed in the I and MP regions (~1,500 cells/mm^2^), while the D region exhibited lower nucleostemin cell density (~750 cells/mm^2^) (Fig. 7).

**Fig. 8:**
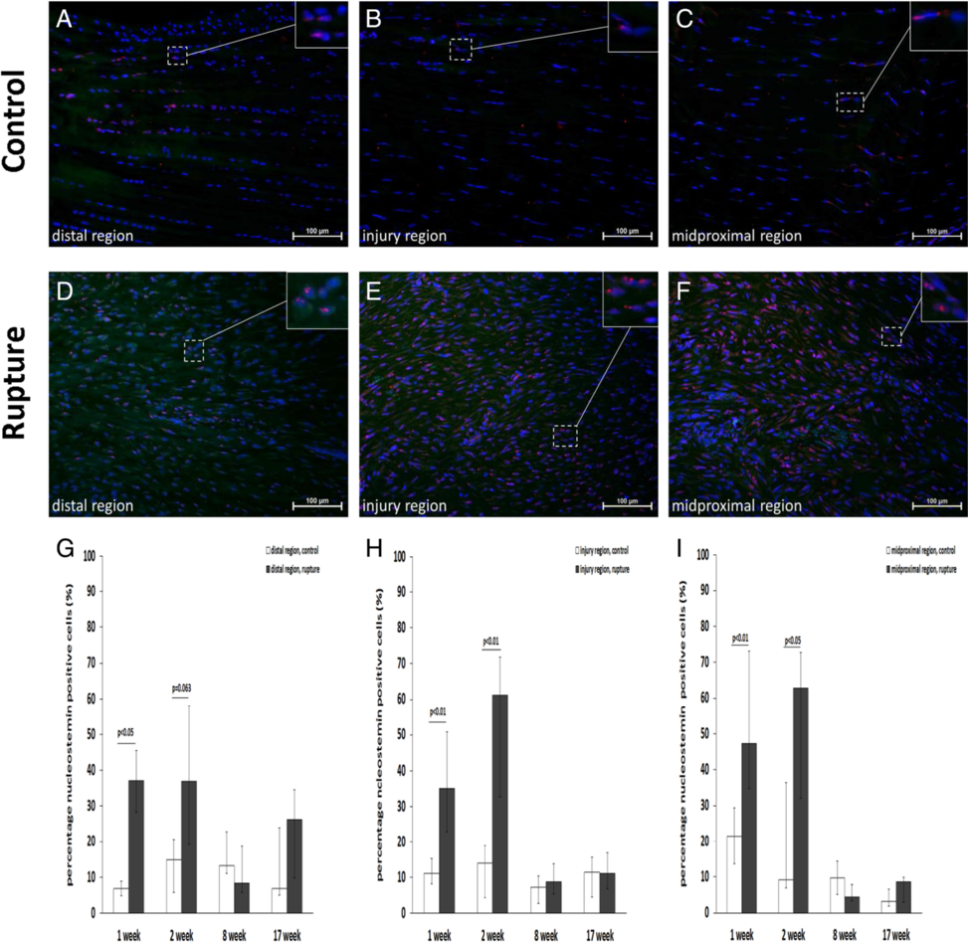
Nucleostemin percentage and presence in response to tendon rupture. Images showing immunofluorescence staining of the stem cell marker, nucleostemin (red), in control (**a**-**c**) and ruptured (**d**-**f**) rat Achilles tendon at week two post-rupture. Diagrams showing the percentage of nucleostemin-positive cells in the distal (**g**), injury (**h**) and midproximal (**i**) regions at one, two, eight and 17 weeks post-rupture. A total of six animals were evaluated at each time point. The *p* values in the figure represent comparisons between the control (left leg *n* = 6) and ruptured (right leg *n* = 6) tendon at each time point. Values are expressed as the median with IQR as error bars

At weeks one and two post-rupture, the percentage of nucleostemin-positive cells in the different regions thus equalled approximately 40 to 60 % of all cells (Fig. 8). At weeks eight and 17 post-rupture, however, the density of nucleostemin-positive cells returned to approximately the same level as in the control tendons (Fig. 8), which equals around 5-10 % of the total cell number.

### Regional differences in nucleostemin-positive cell percentage in injured tendons at week 17

**Fig. 9:**
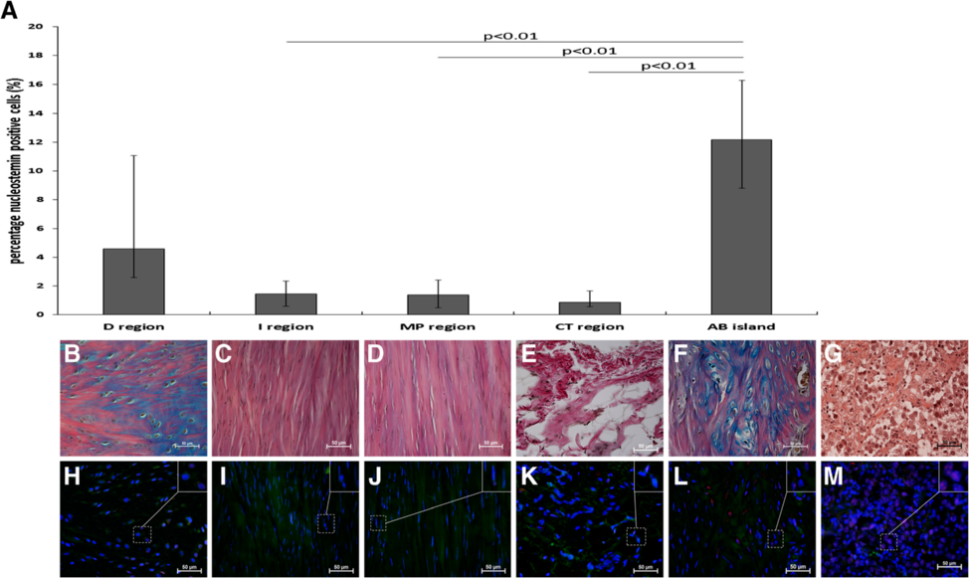
Nucleostemin percentage and presence at different locations in healing tendon at week 17. The percentage of nucleostemin-positive cells was calculated in five different regions from ruptured (right leg; *n* = 6) tendons at week 17. The diagram (**a**) shows a significantly higher percentage of nucleostemin-positive cells in regions with high Alcian blue content (AB islands) compared with I, MP and CT regions. Values are expressed as the median with IQR as error bars. Images showing histological staining (**b**-**f**) and nucleostemin (red) staining (**h**-**l**) in the different regions of the tendons. Positive control tissue for nucleostemin was human seminoma tissue (**g**,**m**)

Significant differences in the nucleostemin-positive cell percentage were found when comparing all regions in injured tendons at 17 weeks post-injury (p < 0.01). The percentage of nucleostemin-positive cells was specifically higher in Alcian blue islands compared with the I, MP and CT regions (p < 0.01 for all), whereas no significant difference was found when compared with the D region (Fig. 9).

## Discussion

The injured rat Achilles tendon displays structural changes, with a large increase in cell proliferation throughout the tendon length. In particular, the three times higher cell density in the I and MP regions at all observed time points (up to 17 weeks) suggests a prolonged healing process after Achilles tendon rupture, which may involve the disrupted mechanical transmission of force. Moreover, the numbers of stem/progenitor cells not only increase in the injured region but are also identified as upregulated numbers throughout the tendon length, implying a non-specific general role in the healing process. Most of these stem/progenitor cells at this time point (1 and 2 weeks post-injury) were found to have a nucleostemin-positive phenotype. Nucleostemin is expressed predominantly in primitive undifferentiated cells and has been used as a stem/progenitor marker in several tissues [14–16]. Earlier studies have shown that nucleostemin is involved in proliferation processes, either in stem/progenitor cells or in cancer cells [17, 18]. In the context of the ruptured Achilles tendon, nucleostemin-positive cells increase, with an increment of up to 40-60 % of the total cell number in the early healing phase, suggesting that they play an important role in the cell proliferation of a healing rat tendon. A similar finding has been reported in mouse myocardium, where the induction of nucleostemin was seen in cardiomyocytes following cardiomyopathic injury [15]. Furthermore, the forced overexpression of nucleostemin in cultured cardiac stem cells significantly increased their cell proliferation and the authors proposed the potential use of nucleostemin as a molecular interventional tool in cardiac pathophysiological conditions [15]. Nucleostemin could possibly also be used in interventional strategies for tendon regeneration.

The specific finding of a few Oct 3/4-positive cells within the injured tendons, mainly in the I region, suggests a more precise role for these cells in this area of the healing rat tendon. The source of these stem/progenitor cells may be the tissues surrounding the tendon, as more Oct 3/4-positive cells were identified in the loose connective tissue surrounding the tendon (peritendon) and in the MTJ region in both control and ruptured tendons. Our observation of Oct 3/4 in the same location as Dynamin 2, a large GTPase recently found to stimulate tenocyte migration [19], furthermore indicates the migration potential of these cells, which was not seen with the nucleostemin-positive cells. Several reports indicate that the stem/progenitor cell populations in and around the tendon are mixed [8, 9, 11] and this probably also applies to the Oct 3/4-positive and nucleostemin-positive cell population in this study, as none of these markers specifically detect only a single homogeny variant of stem/progenitor cells. It is well established that tendon healing occurs through both intrinsic and extrinsic healing [20] and Cadby et al. found that the peritenon stem/progenitor cells appeared to have a faster migration rate and a higher expression of mesenchymal stem cell (MSC) markers [8]. Furthermore, Mienaltowski et al. [12] provided evidence that peritendon progenitors secrete a factor/factors which stimulates/stimulate tenogenic differentiation in tendon-proper progenitors and tenocytes, suggesting a trophic role for the peritendon progenitors.

Whether the Oct 3/4-positive cells found inside the injury region, in contrast to the nucleostemin-positive cells, are peritendon-derived MSCs needs be further evaluated, but the migration of endogenous stem/progenitor cells in tendon repair tissue has recently been shown in rat patellar tendon [11]. The MSCs have the potential to act as both immunosuppressors and regulators of proliferation and extracellular matrix production in damaged tissue [21]. The Oct 3/4-positive stem/progenitor cells observed inside the I region may thus perform a wider regulatory role, such as the orchestration of both inflammatory and proliferative processes during tendon repair. Nucleostemin-positive stem/progenitor cells, on the other hand, may have a general mission to increase the cell population by activating cell proliferation.

Moreover, it was shown that the D region had a smaller increase in both nucleostemin-positive cells and overall cell density at the early time points post-tendon rupture when compared with the I and MP regions. This was surprising, as, in a previous study, it was shown that the D region in rat Achilles tendon had a higher resident pool of stem/progenitor cells compared with the MP region [10]. This was subsequently confirmed in a study of rat patellar tendon by Tan et al. [11]. By 17 weeks post-rupture, we found that the total cell density in the D region of ruptured tendons had returned to normal levels, unlike the I and MP regions, which still had significantly higher cell densities compared with the control tendons. Whether the larger pool of resident (intrinsic) stem/progenitor cells in the D region in rat Achilles tendon causes this region to require less external (extrinsic) assistance in healing processes after a mid-rupture needs to be studied in more detail. It should be noted that the number of nucleostemin-positive cells in the D region in the present study did not differ from the numbers found in the I and MP regions of control tendons in our previous report [10]. However, in the present study, the contralateral left tendon was used as a control and the possibility that some bilateral neuronal effect affected the contralateral tendon, as seen in both rat and rabbit tendon, cannot be excluded [22, 23].

When the tendon healing had entered the remodelling phase (8–17 weeks post-rupture), the number of nucleostemin-positive cells declined to a level comparable to the levels in control tendons, an indication that the cells had lost their stem/progenitor phenotype and entered into a more differentiated state in which proliferation had diminished. The constant high levels of total cells found in the I and MP regions of the tendon compared with the D region at these time points indicate that the remodelling phase in these areas has a heterogenic healing pattern and may reflect less demand in the D region for remodelling processes such as increased collagen I production [1, 24]. At these time points, we furthermore found residing islands of high proteoglycan content (Alcian blue staining), mainly situated in the I and MP regions. They contained cells that were histologically more consistent with a chondrogenic profile and may therefore create a potential disturbance in rebuilding the tendon-proper matrix, leading to biomechanically inferior tissue. Significantly higher proportions of nucleostemin-positive cells were found in these regions when compared with regions with no visible Alcian blue staining, indicating a higher proliferation state and a possible regeneration error in these areas. Interestingly, a recent report by Asai et al. [25] found that progenitor cells from injured tendons had greater chondrogenic potential than progenitors from uninjured tendons and may therefore contribute to chondrogenic degeneration in tendon healing. When transplanted into an injured tendon, a subpopulation of progenitor cells with a CD105-negative phenotype was found to be more likely to induce chondrogenic degeneration at the edges of the injured region compared with progenitors at the centre of the injury. The authors suggested that differences in mechanical forces in these regions may explain the regulation when obtaining tendon-proper tissue regeneration or chondrogenic tissue degeneration. Whether different mechanical influences are responsible for the histological differences in the Alcian blue islands seen in the present study remains to be clarified, but this could provide valuable information when it comes to acquiring a better insight into ways of retaining a tendon with more optimal mechanical function after a rupture.

This study is not without its limitations. First, we did not study each day during the first week after an Achilles tendon rupture and no data on exactly when stem/progenitor cells emerge in the tendon rupture together with the first inflammatory response are therefore available. Second, the transcription factor, Oct4, is able to encode two functional isoforms, Oct4A and Oct4B, whereas only the Oct4A isoform is able to sustain stem cell properties [26]. In addition, there are several homologous pseudo-genes which may give false-positive results [27]. For this reason, we used the monoclonal antibody, sc-5279 (Santa Cruz), which specifically recognises the Oct4A isoform, excluding any false interpretation of Oct4B staining [27].

## Conclusions

In conclusion, a substantial cell increase, combined with a high proportion of stem/progenitor cells, was seen throughout the length of the Achilles tendon after a mid-tendon rupture. The large number of stem/progenitor cells with nucleostemin expression could be of general importance in proliferation maintenance in all tendon regions, while the few stem/progenitor cells with Oct 3/4 expression, mainly seen inside the I region, may perform a more specific regulatory role in the healing tendon. Moreover, the distal tendon region appears to require less extrinsic healing assistance. Residing proteoglycan-rich areas containing clusters of more chondrocyte-like cells, in combination with higher proportions of nucleostemin-positive cells, may be an indication of a specific co-ordination problem in the healing processes, which might have a negative impact on the mechanical function after an Achilles mid-tendon rupture.
